# Disparities in Survival and Comorbidity Burden Between Asian and Native Hawaiian and Other Pacific Islander Patients With Cancer

**DOI:** 10.1001/jamanetworkopen.2022.26327

**Published:** 2022-08-12

**Authors:** Kekoa Taparra, Vera Qu, Erqi Pollom

**Affiliations:** 1 Department of Radiation Oncology, Stanford University, Palo Alto, California; 2 Palo Alto Veterans Affairs Hospital, Palo Alto, California

## Abstract

**Importance:**

Improper aggregation of Native Hawaiian and other Pacific Islander individuals with Asian individuals can mask Native Hawaiian and other Pacific Islander patient outcomes. A comprehensive assessment of cancer disparities comparing Asian with Native Hawaiian and other Pacific Islander populations is lacking.

**Objective:**

To compare comorbidity burden and survival among East Asian, Native Hawaiian and other Pacific Islander, South Asian, and Southeast Asian individuals with non-Hispanic White individuals with cancer.

**Design, Setting, and Participants:**

This retrospective cohort study used a national hospital-based oncology database enriched with Native Hawaiian and other Pacific Islander and Asian populations. Asian, Native Hawaiian and other Pacific Islander, and White individuals diagnosed with the most common cancers who received treatment from January 1, 2004, to December 31, 2017, were included. Patients younger than 18 years, without pathologic confirmation of cancer, or with metastatic disease were excluded. Data were analyzed from January to May 2022.

**Main Outcomes and Measures:**

The primary end points were comorbidity burden by Charlson-Deyo Comorbidity Index and overall survival (OS).

**Results:**

In total, 5 955 550 patients were assessed, including 60 047 East Asian, 11 512 Native Hawaiian and other Pacific Islander, 25 966 South Asian, 42 815 Southeast Asian, and 5 815 210 White patients. The median (IQR) age was 65 (56-74) years, median (IQR) follow-up was 58 (30-96) months, and 3 384 960 (57%) were women. Patients were predominantly from metropolitan areas (4 834 457 patients [84%]) and the Southern United States (1 987 506 patients [34%]), with above median education (3 576 460 patients [65%]), and without comorbidities (4 603 386 patients [77%]). Cancers included breast (1 895 351 patients [32%]), prostate (948 583 patients [16%]), kidney or bladder (689 187 patients [12%]), lung (665 622 patients [11%]), colorectal (659 165 patients [11%]), melanoma (459 904 patients [8%]), endometrial (307 401 patients [5%]), lymphoma (245 003 patients [4%]), and oral cavity (85 334 patients [1%]) malignant neoplasms. Native Hawaiian and other Pacific Islander patients had the highest comorbidity burden (adjusted odds ratio [aOR], 1.70; 95% CI, 1.47-1.94) compared with Asian and White groups. Asian patients had superior OS compared with White patients for most cancers; only Southeast Asian patients with lymphoma had inferior survival (adjusted hazard ratio [aHR], 1.26; 95% CI, 1.16-1.37). In contrast, Native Hawaiian and other Pacific Islander patients demonstrated inferior OS compared with Asian and White patients for oral cavity cancer (aHR, 1.56; 95% CI, 1.14-2.13), lymphoma (aHR, 1.35; 95% CI, 1.11-1.63), endometrial cancer (aHR, 1.30; 95% CI, 1.12-1.50), prostate cancer (aHR, 1.29; 95% CI, 1.14-1.46), and breast cancer (aHR, 1.09; 95% CI, 1.00-1.18). No cancers among Native Hawaiian and other Pacific Islander patients had superior OS compared with White patients.

**Conclusions and Relevance:**

In this cohort study, compared with White patients with the most common cancers, Asian patients had superior survival outcomes while Native Hawaiian and other Pacific Islander patients had inferior survival outcomes. Native Hawaiian and other Pacific Islander patients had significantly greater comorbidity burden compared with Asian and White patients, but this alone did not explain the poor survival outcomes. These results support the disaggregation of these groups in cancer studies.

## Key Points

**Question:**

Are there differences in survival and comorbidity burden among East, South, and Southeast Asian patients vs Native Hawaiian and other Pacific Islander patients with nonmetastatic cancer?

**Findings:**

This cohort study among 5 955 550 patients found that while Asian patients with cancer had significantly superior survival compared with non-Hispanic White patients, Native Hawaiian and other Pacific Islander patients with most cancers did not. Unlike Asian patients, Native Hawaiian and other Pacific Islander patients with cancer had significantly higher comorbidity burden compared with non-Hispanic White patients.

**Meaning:**

These findings suggest that data for Native Hawaiian and other Pacific Islander patients should not be aggregated with Asian patients, as this may mask Native Hawaiian and other Pacific Islander disparities with significant implications for health policy.

## Teaser

This cohort study compares comorbidity burden and overall survival among Asian patients vs Native Hawaiian or other Pacific Islander patients compared with non-Hispanic White patients.

## Introduction

Native Hawaiian and other Pacific Islander individuals are frequently aggregated with Asian individuals or excluded altogether in medical research.^1,2^ This is despite federal guidelines separating data for Native Hawaiian and other Pacific Islander individuals from data for Asian individuals.^3^ Native Hawaiian and other Pacific Islander individuals share ancestry from nearly 30 island nations across Melanesia, Micronesia, and Polynesia and experience dissimilar health disparities, including higher rates of diabetes, obesity, asthma, and cardiovascular diseases, compared with Asian individuals.^2^ Moreover, the Asian population is not a monolithic group, and few studies have investigated the differences in clinical outcomes among East, South, and Southeast Asian patients. This paucity of medical research masks existing disparities, which can influence public policy and funding allocation.^2^

Cancer is the leading cause of death for the Asian, Native Hawaiian, and other Pacific Islander population in the United States.^4^ However, most studies show that the aggregate group has superior cancer outcomes compared with non-Hispanic White (hereafter, *White*) individuals.^5,6,7,8,9,10^ Reports enriched with Native Hawaiian and other Pacific Islander populations suggest inferior survival outcomes among Native Hawaiian and other Pacific Islander patients with cancer.^11,12,13^ To our knowledge, there is no comprehensive report on disaggregated Native Hawaiian and other Pacific Islander cancer disparities on a national scale. Thus, the objective of this study was to elucidate the heterogeneity in comorbidity burden and overall survival (OS) among a large cohort of Asian and Native Hawaiian and other Pacific Islander patients with cancer.

## Methods

### Study Design

This cohort study was deemed exempt from review and informed consent by the Stanford University institutional review board because data were deidentified and publicly available. This study followed the Strengthening the Reporting of Observational Studies in Epidemiology (STROBE) reporting guideline.

### Setting

Asian, Native Hawaiian, and other Pacific Islander patients with cancer in the United States between January 1, 2004, and December 31, 2017, were evaluated. Data were collected from the National Cancer Database (NCDB), a hospital-based, comprehensive data set that captures more than 70% of all newly diagnosed malignancies in the United States.^14^

### Participants

The 9 most common malignant neoplasms in the United States evaluated were breast, lung, prostate, colorectal, kidney or bladder, lymphoma, melanoma, endometrial, and oral cavity cancers.^15^ Inclusion criteria included Asian, Native Hawaiian and other Pacific Islander, and White individuals aged at least 18 years with pathologically confirmed nonmetastatic disease. Self-reported Asian and Native Hawaiian and other Pacific Islander patients were disaggregated into 4 populations: East Asian (Chinese, Japanese, and Korean), Native Hawaiian and other Pacific Islander (Native Hawaiian, Micronesian, Chamorro, Guamanian, Polynesian, Tahitian, Sāmoan, Tongan, Melanesian, Fiji Islander, New Guinean, and other Pacific Islander), South Asian (Indian and Pakistani), and Southeast Asian (Cambodian, Filipino, Hmong, Laotian, Kampuchean, Thai, and Vietnamese). White patients were used as a referent control group.

### Variables

The primary end points for comparison were OS and Charlson-Deyo Comorbidity Index (CCI). OS was defined as months from initial diagnosis to date of last contact or death. CCI is a weighted index that accounts for patient comorbidity number and severity.^14^

### Data Sources and Measurements

Patient covariables included age (years), miles between patient zip code or city centroid and the hospital, zip code–based income (above vs below median), rurality (urban or rural vs metropolitan), zip code–based education (above vs below median), insurance status, comorbidity burden (CCI ≤2, indicating lower comorbidity burden, vs CCI ≥3, indicating higher comorbidity burden), facility type, and US region. Socioeconomic status variables were estimated based on census-level data. Patients with missing data were included and reported accordingly.

### Statistical Analysis

Patient characteristics were reported as number and frequency for categorical variables and median (IQR) for continuous variables. OS was analyzed with Kaplan-Meier estimates and compared using log-rank tests. Multivariable logistic regression assessed high CCI comorbidity burden with adjusted odds ratios (aORs) and multivariable Cox proportional hazard assessed OS with adjusted hazard ratios (aHRs), both with 95% CIs. All regression analyses controlled for patient and disease confounding characteristics to limit bias. Both the logistic regression outcome and covariable for CCI were coded as lower vs higher comorbidity burden. Proportional hazards assumptions were tested. Covariables that violated the proportionality assumptions were fit to the model with stratification. All tests were 2-tailed with statistical significance set at *P* = .05 unless stated otherwise. All statistical analyses were conducted using R statistical software version 4.0.3 in RStudio version 1.3.1093 (R Project for Statistical Computing). Data were analyzed from January to May 2022.

## Results

### Patient Characteristics

Of 11 185 375 patients diagnosed with the 9 most common cancers, 5 955 550 patients met inclusion criteria, including 60 047 East Asian patients, 11 512 Native Hawaiian and other Pacific Islander patients, 25 966 South Asian patients, 42 815 Southeast Asian patients, and 5 815 210 White patients (Table). Median (IQR) age was 65 (56-74) years. Overall, patients were predominantly women (3 384 960 patients [57%]), metropolitan (4 834 457 patients [84%]), from the Southern US (1 987 506 patients [34%]), with above median education (3 576 460 patients [65%]), and without comorbidities (4 603 386 patients [77%]). The proportion of cancers were 1 895 351 patients (32%) with breast cancer, 948 583 patients (16%) with prostate cancer, 689 187 patients (12%) with kidney or bladder cancer, 665 622 patients (11%) with lung cancer, 659 165 patients (11%) with colorectal cancer, 459 904 patients (8%) with melanoma, 307 401 patients (5%) with endometrial cancer, 245 003 patients (4%) with lymphoma, and 85 334 patients (1%) with oral cavity cancer. Cancer staging was 834 131 patients (14%) with stage 0, 2 393 323 patients (40%) with stage I, 1 757 616 patients (30%) with stage II, and 970 480 patients (16%) with stage III (Table).

**Table.** Demographic and Clinical Characteristics of Asian, Native Hawaiian and Other Pacific Islander, and Non-Hispanic White Patients Diagnosed With the 9 Most Common Cancers in the United States

Characteristic	Patients, No. (%)						
	Overall (N = 5 955 550)	East Asian (n = 60 047)	Native Hawaiian and other Pacific Islander (n = 11 512)	South Asian (n = 25 966)	Southeast Asian (n = 42 815)	Non-Hispanic White (n = 5 815 210)
Age, median (IQR), y	65 (56-74)	63 (52-74)	60 (51-69)	60 (49-68)	61 (52-70)	65 (56-74)
Sex						
Men	2 570 590 (43)	20 323 (34)	3728 (32)	9467 (36)	13 650 (32)	2 523 422 (43)
Women	3 384 960 (57)	39 724 (66)	7784 (68)	16 499 (64)	29 165 (68)	3 291 788 (57)
Year of Diagnosis						
2004-2010	2 809 277 (47)	26 492 (44)	4678 (41)	9684 (37)	18 075 (42)	2 750 348 (47)
2011-2017	3 146 273 (53)	33 555 (56)	6834 (59)	16 282 (63)	24 740 (58)	3 064 862 (53)
Distance To Hospital						
Median (IQR), miles	10 (5-24)	6 (3-11)	9 (4-18)	8 (4-14)	7 (4-12)	10 (5-24)
Unknown	456 494	3475	1155	1970	3667	446 227
Income						
Above median	1 890 736 (34)	10 007 (18)	2023 (20)	3911 (16)	7646 (20)	1 867 149 (35)
Below median	3 601 061 (66)	46 584 (82)	8330 (80)	20 076 (84)	31 500 (80)	3 494 571 (65)
Unknown	463 753	3456	1159	1979	3669	453 490
Rurality						
Metropolitan	4 834 457 (84)	57 320 (98)	10 404 (92)	24 505 (98)	41 042 (98)	4 701 186 (84)
Urban or rural	931 855 (16)	1320 (2)	881 (8)	503 (2)	1010 (2)	928 141 (16)
Unknown	189 238	1407	227	958	763	185 883
Education						
Above median	3 576 460 (65)	36 113 (64)	6552 (63)	16 416 (68)	19 299 (49)	3 498 080 (65)
Below median	1 917 985 (35)	20 486 (36)	3801 (37)	7577 (32)	19 851 (51)	1 866 270 (35)
Unknown	461 105	3448	1159	1973	3665	450 860
Insurance Status						
Private	2 652 888 (45)	29 255 (49)	5916 (52)	14 328 (57)	22 838 (54)	2 580 551 (45)
Medicaid or Medicare	3 106 095 (53)	28 615 (48)	5163 (45)	9555 (38)	18 143 (43)	3 044 619 (53)
Uninsured	95 394 (2)	1497 (3)	287 (3)	1424 (6)	1342 (3)	90 844 (2)
Unknown	101 173	680	146	659	492	99 196
Facility Type						
Academic	1 907 263 (33)	27 443 (48)	4692 (44)	11 404 (48)	15 293 (38)	1 848 431 (33)
Community	533 381 (9)	6250 (11)	1461 (14)	2106 (9)	5005 (12)	518 559 (9)
Comprehensive community cancer program	2 533 536 (44)	18 866 (33)	3521 (33)	7752 (33)	16 051 (40)	2 487 346 (44)
Integrated	791 073 (14)	5014 (9)	1097 (10)	2460 (10)	4246 (10)	778 256 (14)
Unknown	190 297	2474	741	2244	2220	182 618
US Region						
Northeast	1 285 445 (22)	13 888 (24)	855 (8)	8544 (36)	5349 (13)	1 256 809 (22)
Midwest	1 549 707 (27)	3654 (6)	806 (8)	4700 (20)	3913 (10)	1 536 634 (27)
South	1 987 506 (34)	5856 (10)	1607 (15)	6475 (27)	5884 (14)	1 967 684 (35)
West	942 595 (16)	34 175 (59)	7503 (70)	4003 (17)	25 449 (63)	871 465 (15)
Unknown	190 297	2474	741	2244	2220	182 618
Cancer stage						
0	834 131 (14)	9551 (16)	1369 (12)	3735 (14)	6315 (15)	813 161 (14)
I	2 393 323 (40)	23 087 (38)	4500 (39)	9784 (38)	15 791 (37)	2 340 161 (40)
II	1 757 616 (30)	17 357 (29)	3548 (31)	8345 (32)	12 716 (30)	1 715 650 (30)
III	970 480 (16)	10 052 (17)	2095 (18)	4102 (16)	7993 (19)	946 238 (16)
Cancer type						
Breast	1 895 351 (32)	26 059 (43)	4955 (43)	11 987 (46)	19 657 (46)	1 832 693 (32)
Prostate	948 583 (16)	7181 (12)	1550 (13)	3954 (15)	5223 (12)	930 675 (16)
Kidney or bladder	689 187 (12)	4879 (8)	924 (8)	2162 (8)	2502 (6)	678 720 (12)
Lung	665 622 (11)	6140 (10)	951 (8)	1323 (5)	4053 (10)	653 155 (11)
Colorectal	659 165 (11)	9545 (16)	1172 (10)	2692 (10)	5895 (14)	639 861 (11)
Melanoma	459 904 (8)	400 (1)	199 (2)	149 (1)	224 (1)	458 932 (8)
Endometrial	307 401 (5)	2839 (5)	1221 (11)	1574 (6)	2969 (7)	298 798 (5)
Lymphoma	245 003 (4)	2295 (4)	416 (4)	1382 (5)	1997 (5)	238 913 (4)
Oral cavity	85 334 (1)	709 (1)	124 (1)	743 (3)	295 (1)	83 463 (1)
CCI						
0	4 603 386 (77)	50 230 (84)	8317 (72)	20 777 (80)	34 430 (80)	4 489 632 (77)
1	992 629 (17)	7878 (13)	2298 (20)	4218 (16)	6614 (15)	971 621 (17)
2	258 669 (4)	1404 (2)	630 (6)	691 (3)	1237 (3)	254 707 (4)
≥3	100 866 (2)	535 (1)	267 (2)	280 (1)	534 (1)	99 250 (2)
Follow-up, median (IQR), mo	58 (30-96)	59 (31-97)	56 (30-93)	55 (31-89)	59 (32-95)	58 (30-96)
Mortality rate	1 714 045 (29)	12 010 (20)	2539 (22)	3452 (13)	8232 (19)	1 687 812 (29)

Abbreviation: CCI, Charlson-Deyo Comorbidity Index.

### Comorbidities Among Asian and Native Hawaiian and Other Pacific Islander Populations

**Figure 1.**
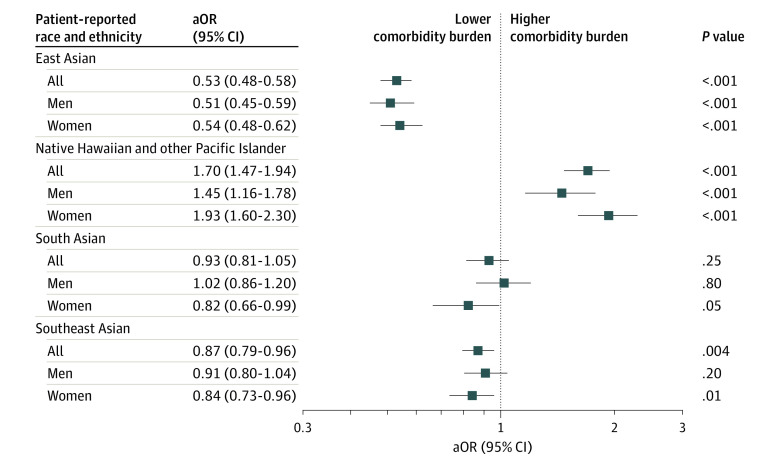
Multivariable Logistic Regression of Comorbidity Burden for Asian and Native Hawaiian and Other Pacific Islander Patients With Cancer Comorbidity burden was measured by Charlson-Deyo Comorbidity Index (low: ≤2 vs high: ≥3) for patients with the most common cancers in the United States. The reference group is non-Hispanic White patients, represented by the dashed line. All regressions were adjusted for patient age, sex (overall group only), year of diagnosis, distance from hospital based on zip code, income based on zip code, education based on zip code, insurance status, cancer stage, facility type, and facility location. aOR indicates adjusted odds ratio.

Compared with White patients, East Asian patients were the least likely to have multiple comorbidities (aOR, 0.53; 95% CI, 0.48-0.58) (Figure 1). South and Southeast Asian women were also less likely to have multiple comorbidities. In contrast, Native Hawaiian and other Pacific Islander patients with cancer had significantly increased comorbidity burden compared with White patients (men: aOR, 1.45; 95% CI, 1.16-1.78; women: aOR, 1.93; 95% CI, 1.60-2.30; overall: (aOR, 1.70, 95% CI, 1.47-1.94).

### Survival Among Asian and Native Hawaiian and Other Pacific Islander Populations

**Figure 2.**
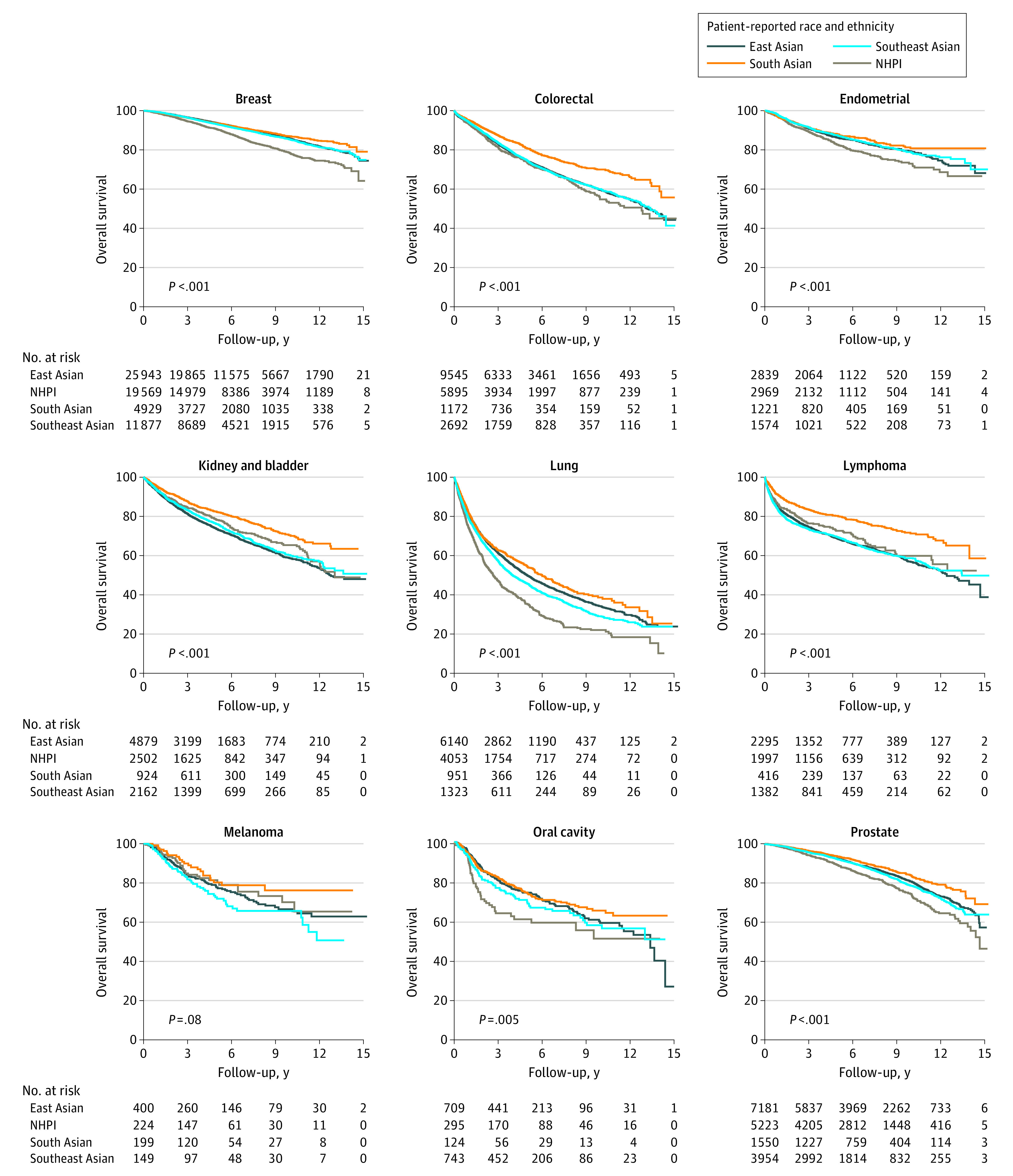
Kaplan-Meier Estimates for Overall Survival Probability for Asian and Native Hawaiian and Other Pacific Islander (NHPI) Patients With Cancer

Overall median (IQR) follow-up was 58 (30-96) months. The mortality rate was 20% among East Asian patients, 22% among Native Hawaiian and other Pacific Islander patients, 13% among South Asian patients, and 19% among Southeast Asian patient (Table). Figure 2 displays unadjusted Kaplan Meier survival curves.

**Figure 3.**
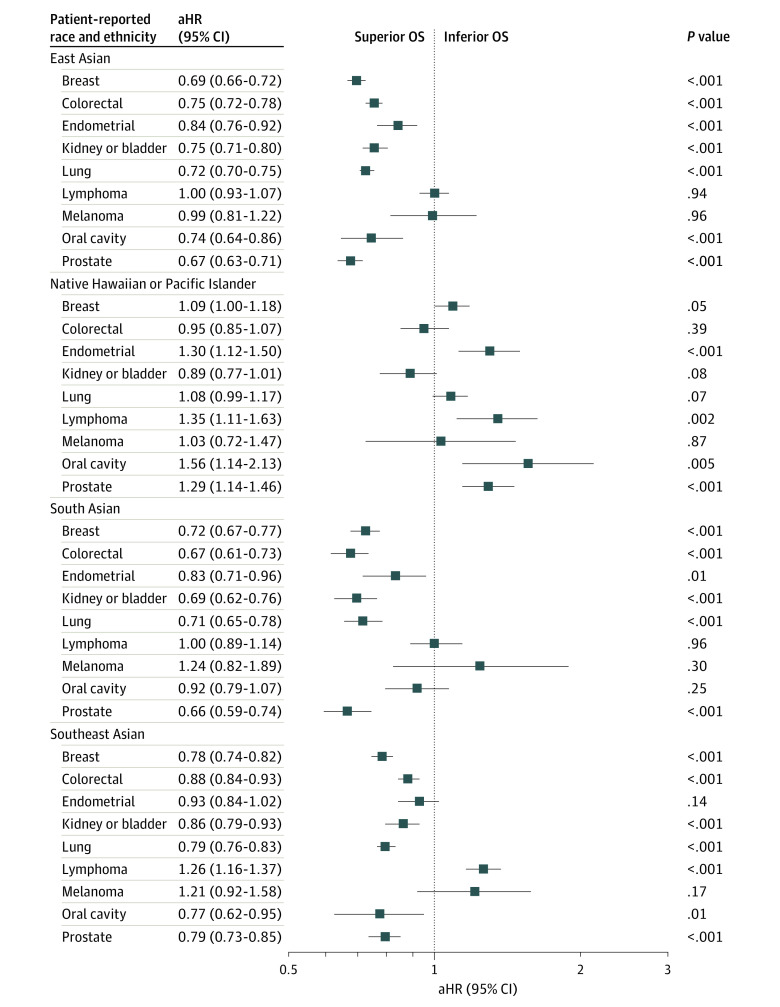
Multivariable Cox Proportional Hazard Regression of Overall Survival (OS) for Asian and Native Hawaiian and Other Pacific Islander Patients With Cancer The reference group is non-Hispanic White patients, represented by the dashed line. All regressions were adjusted for patient age sex (except prostate, endometrial, and breast cancers), year of diagnosis, distance from hospital based on zip code, income based on zip code, education based on zip code, insurance status, cancer stage, medical comorbidities (Charlson-Deyo Comorbidity Index ≤2 vs ≥3), facility type, and facility location. aHR indicates adjusted hazard ratio.

After adjusting for confounders, including comorbidity burden, most Asian groups (19 of 27 groups [70%]) demonstrated superior OS compared with White patients: 7 of 9 cancers among East Asian patients, 6 of 9 cancers among South Asian patients, and 6 of 9 cancers among Southeast Asian patients (Figure 3). The only Asian group with inferior OS compared with White patients was Southeast Asian patients with lymphoma (aHR, 1.26; 95% CI, 1.16-1.37). Survival was not different for melanoma between White patients and any Asian group.

In contrast, most Native Hawaiian and other Pacific Islander patients with cancer demonstrated inferior survival compared with White patients, including oral cavity (aHR, 1.56; 95% CI, 1.14-2.13), lymphoma (aHR, 1.35; 95% CI, 1.11-1.63), endometrial (aHR, 1.30; 95% CI, 1.12-1.50), prostate (aHR, 1.29; 95% CI, 1.14-1.46), and breast (aHR, 1.09; 95% CI, 1.00-1.18) cancers (Figure 3). Native Hawaiian and other Pacific Islander patients with lung cancer did not have worse OS compared with White patients (aHR, 1.08; 95% CI, 0.99-1.17). There were no cancers among Native Hawaiian and other Pacific Islander patients that demonstrated superior OS compared with the White group.

## Discussion

In this large cohort study, we found striking differences in comorbidity burden and survival outcomes between the commonly aggregated Asian and Native Hawaiian and other Pacific Islander populations with cancer. All East, South, and Southeast Asian populations had significantly superior or similar OS compared with White patients with cancer. In contrast, Native Hawaiian and other Pacific Islander patients had inferior OS across most cancers. This underscores the danger of aggregating Native Hawaiian and other Pacific Islander patients with Asian patients.

With more than 11 000 Native Hawaiian and other Pacific Islander patients with cancer included, this is likely the largest and most comprehensive study to evaluate cancer disparities among Native Hawaiian and other Pacific Islander patients in the United States. Our findings directly contrast with prior studies using similar databases reporting superior OS for breast, prostate, lung, endometrial, lymphoma, and oral cavity cancers among aggregated Asian and Native Hawaiian and other Pacific Islander populations compared with White populations.^5,6,7,8,9,10^ The continued aggregation of these 2 groups perpetuates the impression that Native Hawaiian and other Pacific Islander patients with cancer have better outcomes than reality.

The high comorbidity burden among Native Hawaiian and other Pacific Islander patients may impact critical treatment decisions, such as surgical candidacy or consideration for clinical trial enrollment. This may in part translate to inferior cancer outcomes. However, the association inferior OS observed among Native Hawaiian and other Pacific Islander patients persisted even after controlling for comorbidity burden, suggesting that there may be other mediating factors at play. These may include key social determinants of health that may not be accounted for in our models, including cultural diet, lifestyle behaviors, health literacy, racism and discrimination, and access to transportation.^11^

### Limitations

This study has some limitations. Our data are limited by the nature of this retrospective study as well as the NCDB itself, which is hospital-based rather than population-based. The NCDB also lacks clinically important end points, such as patient-reported outcomes, cause of death, and disease-free survival.

## Conclusions

In this cohort study, Native Hawaiian and other Pacific Islander patients with the most common cancers had significantly worse outcomes than East, South, and Southeast Asian patients compared with White patients. These findings highlight the need for Native Hawaiian and other Pacific Islander cancer data disaggregation.
